# Biophysical optimality of the golden angle in phyllotaxis

**DOI:** 10.1038/srep15358

**Published:** 2015-10-16

**Authors:** Takuya Okabe

**Affiliations:** 1 Graduate School of Integrated Science and Technology, Shizuoka University, 3-5-1 Johoku, Hamamatsu 432-8561, Japan

## Abstract

Plant leaves are arranged around a stem axis in a regular pattern characterized by common fractions, a phenomenon known as phyllotaxis or phyllotaxy. As plants grow, these fractions often transition according to simple rules related to Fibonacci sequences. This mathematical regularity originates from leaf primordia at the shoot tip (shoot apical meristem), which successively arise at fixed intervals of a divergence angle, typically the golden angle of 137.5°. Algebraic and numerical interpretations have been proposed to explain the golden angle observed in phyllotaxis. However, it remains unknown whether phyllotaxis has adaptive value, even though two centuries have passed since the phenomenon was discovered. Here, I propose a new adaptive mechanism explaining the presence of the golden angle. This angle is the optimal solution to minimize the energy cost of phyllotaxis transition. This model accounts for not only the high precision of the golden angle but also the occurrences of other angles observed in nature. The model also effectively explains the observed diversity of rational and irrational numbers in phyllotaxis.

Mathematically regular arrangements of plant leaves, flower petals and other homologous organs, a phenomenon known as phyllotaxis, have attracted the attention of biologists, physicists and mathematicians. In the early nineteenth century, Schimper and Braun reported that regularity is expressed by means of common fractions obeying a Fibonacci rule. In a 3/8 phyllotaxis, for instance, every eighth leaf emerges above one below it after three turns of a spiral of successive leaves, so that eight straight ranks are visible along the stem (Fig. 1a). The Fibonacci rule is to add the previous two numbers to obtain the next number. Accordingly, 2/5 is obtained from 1/2 and 1/3 by adding their numerators and denominators, respectively. Although the rule lacks a rational basis, it empirically describes not only the most commonly observed sequence of the phyllotaxis fractions—1/3, 2/5, 3/8, 5/13 and so on—but other rare sequences as well^1^,^2^. A list of these sequences and representative plants is often presented as follows: 1/2 for elm, lime and linden; 1/3 for beech and hazel; 2/5 for oak, cherry, apple, holly and plum; 3/8 for poplar, rose and pear; 5/13 for almond; etc. Some references present willow as 5/13 and others as 3/8 without citing sources^3^,^4^. In fact, the phyllotaxis fraction is not a determined trait of each species but, rather, may vary from one part of a plant to another. This change in fraction is called phyllotaxis transition. The transition on a stem is often very conspicuous, because the denominator of the phyllotaxis fraction represents the number of vertical ranks of leaves. Anatomically, vascular bundles are formed by connecting what are known as the leaf traces of respective leaves, which are readjusted when the phyllotaxis fraction transitions as the plant grows (Fig. 1d)^5^.

In marked contrast to these vertical arrangements, nascent leaves in the bud, or leaf primordia at the shoot apical meristem are more regularly arranged, but their arrangement in no way conforms to a fraction, i.e., a rational number. As a general rule, the divergence angle between successively arising leaves is fixed at the golden angle of 137.5°, i.e., an irrational number^6^. The golden angle is universally observed at the shoot tip of most vascular plants^7^,^8^,^9^,^10^. Approximate explanations for the presence of the golden angle have been attempted since ancient times^7^,^11^,^12^. Recently, plausible numerical models have been put forward to describe the formation of phyllotaxis patterns at the shoot apical meristem^13^,^14^. However, the following questions remain unaddressed in addition to the original problems raised by Schimper and Braun. Why is the innate divergence angle fixed so robustly and accurately? Do the phyllotaxis fraction and its transition, which have been ignored, truly have only a secondary relevance for understanding the accurate phyllotaxis at the shoot tip? Is there any adaptive value of the phyllotaxis phenomenon? This paper presents a model to answer these questions. The model brings a consistent theoretical perspective to multifarious empirical observations that have accumulated in the literature. Specifically, I demonstrate that the golden angle minimizes the energy cost of phyllotaxis transition.

## Results

**Figure 1:**
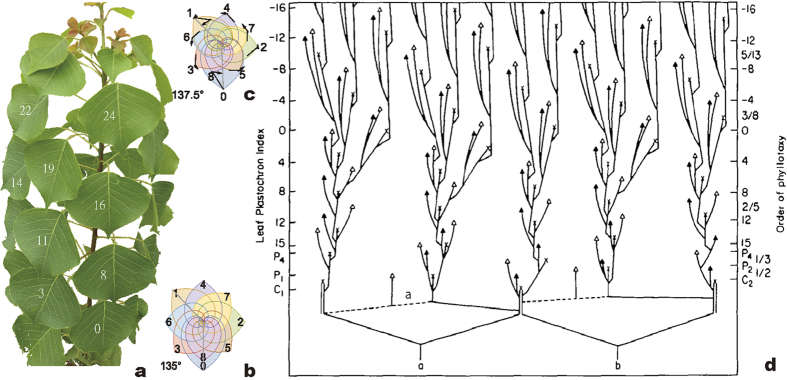
Phyllotaxis transition of a poplar tree. (**a**) A young poplar in a 3/8 phyllotaxis with eight vertical ranks (orthostichies) of leaves. (**b**) Successive leaves on the developed stem make constant angles of 360 × 3/8 = 135°. (**c**) In contrast, the divergence angle at the shoot tip is equal to the golden angle, 137.5°. Therefore, neighbouring leaves form eight winding spirals (parastichies) at the tip. (**d**) Larson’s diagram of leaf traces of a cottonwood poplar (reproduced with permission)^3^. The stem cylinder is displayed as if unrolled and laid flat. The phyllotaxis order progresses from 1/2 through 1/3, 2/5 and 3/8 to 5/13, as denoted by the right vertical axis. Photograph taken by Takuya Okabe.

The pertinent point on which I focus is the empirical fact that phyllotaxis, or the divergence angle, does change between two stages, that is to say, (i) the leaf arrangement at a shoot tip and (ii) the leaf arrangement on a developed stem (cf. p.228f. of ref. 7, p.13 of ref. 15, p.40 of ref. 16). Accordingly, apparent spirals (parastichies) of leaves that are formed at regular intervals of 137.5° (Fig. 1c) are secondarily straightened to either 5, 8, 13, etc. vertical rows (orthostichies) (Fig. 1b) by the accompanying torsion of the elongating stem^6^,^17^.

It is empirically known that both spiral directions occur with equal probabilities to within an accuracy of 1%^18^. Therefore, expressed as a fraction of the total circumference, the divergence angle is restricted from 0 to 1/2 (180°) if measured in the direction of the spiral. In what follows, the divergence angle is expressed according to this convention. I make the basic assumption that the divergence angle at the initial stage (i), *α*_0_, is a heritable trait of an individual plant so that its mean and standard deviation, 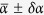, evolve by natural selection to minimize the total cost of twisting the stem, as follows:


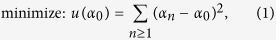


where *α*_*n*_ is the *n*-th divergence angle (between leaves *n* and 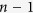) at the mature stage (ii) that depends on *α*_0_. In other words, *α*_*n*_ is a function of *α*_0_ and so is *u*(*α*_0_) (for details, see (5) in Methods and Supplementary Fig. S1). In fact, the former is a rational number (common fraction) approximating the latter (for instance, *α*_*n*_ = 1/3(= 0.333), 2/5(= 0.4), 3/8(= 0.375), 5/13(= 0.385), etc. are rational numbers approximating *α*_0_ = 137.5/360 = 0.382. Rational numbers are relevant because leaves stand in vertical rows). Consequently, the angular shift 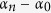 takes a small, definite value and represents the secondary torsion of the stem per leaf. This shift has been measured in practice for normal phyllotaxis (*α*_0_ = 0.382)^6^. Taking into account the statistical variation of *α*_0_, the cost is given by the following:


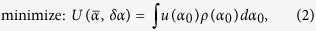


where 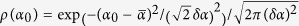 is the normal distribution with mean 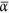 and standard deviation *δα*.

**Figure 2:**
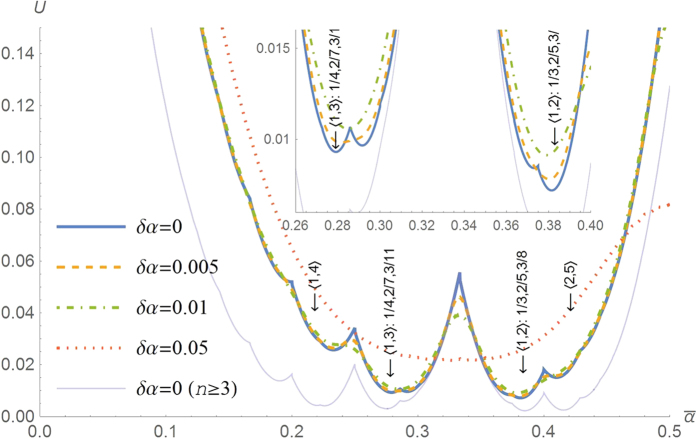
The golden angle minimizes the energy cost of twisting the stem. The energy cost 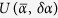 is plotted against the mean divergence 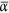 for four values of the standard deviation *δα* = 0, 0.005 (1.8°), 0.01 (3.6°) and 0.05 (18°). The lowest thin curve is obtained by excluding the contributions from the first two leaves (see Methods). The inset shows the absolute minimum at 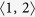: 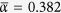 (the golden angle 137.5°) for the main sequence 1/3, 2/5, 3/8, 5/13, 8/21, which is predominant in nature. Indeed, cone scales of the genus *Pinus* normally belong to the main sequence (p. 250 of ref. 2). The subsidiary sequence 1/3, 1/4, 2/7, 3/11, 5/18, corresponding to a local minimum at 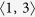: 0.276 (99.5°), also occurs, but rarely. Other exceptional sequences are also observed. See Table 1.

The cost *U* is plotted in Fig. 2 for *δα* = 0, 0.005, 0.01 and 0.05. As the inset shows, *U* has the absolute minimum at the mean value equal to 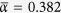, which is indicated by an arrow labelled with “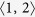: 1/3, 2/5, 3/8”. This value is the golden angle 137.5° giving rise to the main sequence of phyllotaxis *α*_*n*_ = 1/3, 2/5, 3/8, 5/13, 8/21, 13/34. The optimum is reached by decreasing the variance *δα*. Thus, to reduce the cost, the innate divergence angle 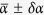 should be converged toward the golden angle through evolution. The cost *U* has local peaks at 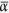 equal to rational numbers (common fractions). On the other side of the most notable peak at 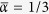 lies a local minimum at 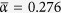 (99.5°), leading to another sequence 1/3, 1/4, 2/7, 3/11, 5/18. As discussed below, this anomaly is occasionally found in many plant species.

## Discussion

The present explanation is free from the drawbacks of previous explanations. I assume that the regular phyllotaxis is a consequence of optimal adaptation. Since ancient times^7^,^11^, almost no models of phyllotaxis that have been put forward have adopted this assumption. Either physical or chemical, these models focus on dynamical mechanisms of how and where leaves arise. Thus, the dynamical models investigate phyllotaxis from the perspective of development and not of evolution. Although these models produce phyllotaxis patterns that are qualitatively similar to many of those found in nature, they have difficulty in explaining the constancy of the divergence angle^7^,^13^,^19^. A common “explanation” that the 137.5° angle is adopted to optimize light falling on individual leaves has not received broad support because light capture (or any function of lateral appendages) is more strongly affected by other factors incidental to phyllotaxis, such as the habitat, leaf width and stalk length, than by the divergence angle of their mutual arrangement^20^. I argue that the key factor lies in the stem. The phyllotaxis transition must entail an energetic cost that varies depending on the degree of change, e.g., in supplying interconnecting vascular tissue to form an integrated network of the vascular system^15^. I present a simple model in which the cost of changing arrangement is represented by the angular shift 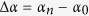 and show that this cost is indeed minimized at the constant divergence angle (*α*_0_) of 137.5°. In answer to the questions posed in the introduction, the innate divergence angle of 137.5° is robust and accurate because it is optimally adapted for the subsequent process of phyllotaxis transition. The cost in Equation (1) is a sum of terms whose minimum lies at a rational value (fraction) *α*_*n*_. Therefore, the phyllotaxis transition, or step-wise change of *α*_*n*_, is essential for explaining an apparently irrational value of the initial divergence angle *α*_0_. If not for phyllotaxis transition, there would be no reason for the phyllotaxis of nascent leaves to be different from the phyllotaxis of mature leaves. The plant that is bound to exhibit the stem phyllotaxis of 3/8 (135°) and 5/13 (138°), depending on circumstances, would be better off adopting a divergence angle of 137° throughout the course of development.

It is an empirical fact^1^,^2^ that the phyllotaxis fraction 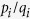 of living plants follows and varies along a sequence given by Fibonacci relations 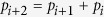 and 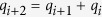. In the phyllotaxis literature, the limit of the sequence,


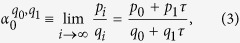


is called the limit divergence angle, where the golden ratio 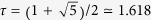 is an irrational number known to ancient Greek mathematicians. The whole sequence 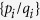 is referred to by the initial number pair 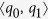 (cf. Supplementary Note). Table 1 presents the limit divergence angles and corresponding sequences for the simplest combinations of *q*_0_ and *q*_1_ along with data on relevant species collected from the literature. Note that the cost in Fig. 2 has local minima at the limit divergence angles 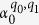 (see Methods). In practice, any sequence other than the main sequence deriving from the golden angle for 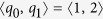 is regarded as anomalous. Typical limit divergence angles have been directly confirmed^9^,^21^.

In addition to the above sequences, Braun reported unusual sequences converging to a member of the main sequence 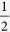, 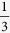, 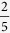 and 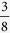, which were applied to several genera of monocotyledons (*Crinum, Aloe,* and *Pandanus*). For instance, the sequence 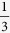, 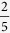, 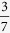, 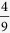, 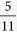, 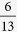 converging to 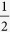 has been found in many species of the genus *Aloe* (pp.305ff. of ref. 2).

Moreover, there are multijugate patterns in that more than one leaf is attached at a node of the stem. The *N*-jugate pattern of *N* leaves at a node is represented by 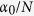, 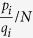, or 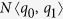. Multijugate spirals are not as common as alternating whorls, which occur in the families Equisetaceae (including *Calamites*) and Lycopodiaceae (including *Lepidodendron*). These families show the greatest variability in phyllotaxis (p. 358 of ref. 2, see below). In accordance with the notation adopted above, alternating whorls may be formally denoted as 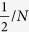 or 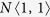, of which well-known distichy and decussate are special cases for 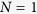 and 2, respectively. A decussate pattern 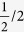 in which successive leaf pairs cross at 90°, is common in the families Caryophyllaceae, Rubiaceae and Dipsacaceae^2^. Despite their apparent similarity, alternating whorls 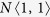 are distinguished from spiralling whorls 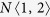 in that the former have bilateral symmetry whereas the latter have chirality, or handedness^8^. The divergence angle of the latter is definitely given by 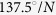 (refs 6,9,22).

The evolutionary trajectories of the divergence angle depend on the genetics of the quantitative trait, which is unknown and most likely polygenetic. The phyllotactic phenotypes are robustly distinct. Moreover, the golden angle of spiral phyllotaxis is so preponderant that it is not even known whether the frequency of the phenotypes has ever followed a continuous variation distribution. It is sufficient here to note that only those individuals with optimal or suboptimal phenotypes are able to survive, which holds true independently of the genetic system. The following observations appear to support to the evolutionary view of the present approach. The variation in phyllotaxis is like any other type of variation: some plants show a tendency to and others a perseverance in their default patterns (Table 2)^2^. Whereas no variation from 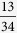 was found among many hundreds of cones of Scots pine *Pinus sylvestris*, there were anomalous patterns in 3% of more than 1000 cones of Norway spruce *Picea abies*, deviating from the normal arrangements of 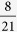, 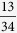 and 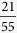. The anomalies comprise 1% of 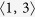 (0.7% of 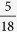 and 0.3% of 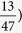 and 2% of the bijugate patterns 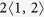 (1.2% of 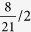, 0.4% of 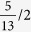 and traces of 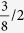 and 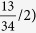. Still notable is the fact that not only individual forests but also individual trees tend to produce the preferred anomalies (pp.389–393 of ref. 2). Therefore, it should be noted that the occurrence rate of anomalous patterns depends not only on the species but also on the geographical area. For the capituli of the sunflower *Helianthus annuus*, which normally belongs to 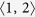, 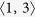 patterns were found in 4%^23^ and 15%^24^ (Table 2). Interestingly, in some species anomalous patterns are standard. *Sedum sexangulare* usually has a 7-ranked pattern with 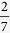 and occasionally changes to a 6-ranked arrangement of alternating trijugate 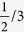, hence the name^2^. The bijugate spiral 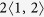 with 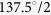 is also generally rare, but there are cases, such as *Cephalotaxus drupacea*^21^,^25^ and *Dipsacus sylvestris*^2^,^4^, in which this spiral is commonly seen (Table 2). These species are noted as showing highly variable patterns (Table 1). The phyllotaxis of *Lepidodendron* fossils is diverse in a very specific manner exhibiting specifically high-order fractions^26^, i.e., 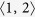 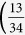, 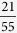, 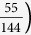, 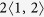 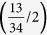, 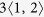 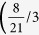, 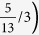, 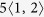 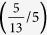, 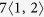 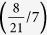, 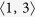 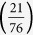, 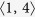 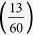, 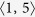 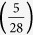, 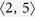 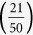, and 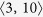 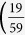, 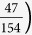. This observation indicates that spiral patterns are more primitive than alternating whorls and that the fine tuning 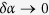 had already occurred before the dominant system 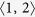 was naturally selected.

Braun categorized all of the conceivable fractions (*p*/*q*) into numbered domains. The domain of *n*- to (*n* + 1)-ranked patterns includes fractions whose values lie between 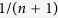 and 1/*n* (delineated by thick vertical lines at 1/*n* in Supplementary Fig. S1). According to Braun, *Sedum acre* varies unalterably in the domain of 2 to 3 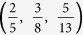, *Sedum sexangulare* persistently belongs to the domain of 3 to 4 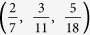, and *Sedum reflexum* stretches over not only both of these domains but also even to the third one 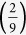. In conifers, *Pinus strobus* shows variations but does not appear to go beyond the main domain of 2 to 3 (cf., the first (asterisk) note in Table 2, pp.389f. of ref. 2). The present model supports the validity of this classification system, as peaks at 1/*n* in the landscape of the energy cost 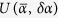 may work as effective barriers.

In this study, I aimed to explain the preponderance of the golden angle in spiral phyllotaxis. It is worth noting that the problem has rarely been formulated as such, because suggestive numbers abound in phyllotaxis. In fact, people tend to be attracted by Fibonacci numbers. Whether the divergence angle is a rational or irrational number has been argued (cf. pp.69ff. of ref. 6; ref. 8; pp.169f. of ref. 27). The present model resolves this problem by using *α*_*n*_ (rational number) on the one hand and *α*_0_ (irrational number) on the other hand and explicates number-related facts of phyllotaxis in a unified manner. This model takes account of the fact that various related fractions (*α*_*n*_) that may occur on different parts of an individual plant originate from one and the same inherited trait *α*_0_. It is reasonable to expect interspecies variations in the variance of *α*_0_ that, however, have not been investigated to the author’s knowledge, though some intraspecies variations have been reported^19^.

## Methods

The phyllotaxis fraction *α*_*n*_ depends not only on the initial divergence angle *α*_0_ but also on the length of leaf traces *l*. The latter is evidenced by the observations showing a significant correlation between *α* and *l*, i.e., higher phyllotactic values are associated with longer traces (p.31 of ref. 15). In general, a large value of *l* represents a densely packed pattern. When *α*_0_ and *l* are constant, the resulting fraction *α* is obtained by a geometrical consideration (Supplementary Figs S1 and S2). For the same initial divergence angle 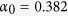 (137.5°), similar patterns with *l* = 4 and 7 result in different patterns of 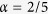 (Supplementary Fig. S2a) and 3/8 (Supplementary Fig. S2b), respectively. In effect, the phyllotaxis pattern of 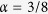 is obtained insofar as 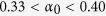 and 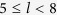. In general, the range of values of *α*_0_ and *l* that result in a given fraction *α* is obtained as delineated in Supplementary Fig. S1. This figure provides a correspondence table of phyllotaxis fraction *α*(*α*_0_, *l*) (ref. 28). It is interesting that Schimper and Braun made use of similar tables to analyse their observations (Tables 1 and 2 of ref. 1; Table L of ref. 2).

In practice, the leaf-trace length *l* varies depending on individual leaves. I assume


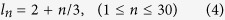


and


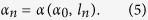


Under these assumptions with *α*_0_ = 0.382 (137.5°), Larson’s diagram of leaf traces (Fig. 1d) is simulated as a theoretical pattern of points 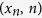 (Supplementary Fig. S3), where the angular position of the *n*-th leaf is given by


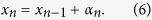


Equations (1), (2), (4) and (5) give the energy cost 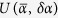 as plotted in Fig. 2.

The present model describes that an irrational number 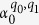 at the shoot tip gives rise to a fraction (rational number) in the sequence 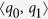 on the mature stem, depending on *l*, i.e.,


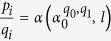


for 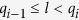 (*i* > 1) and 
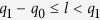
 (*i* = 1). The sequence 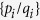 converges to the limit 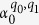 in an oscillatory manner, i.e.,


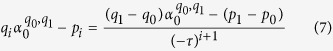


(cf. Supplementary Note). Regular oscillation sets in from 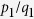 (*i* = 1). The main sequence 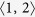 is unique in that it lacks precursory irregularity before 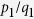 (e.g., 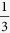 is irregularly inserted in the 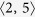 sequence 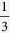 (120°), 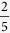 (144°), 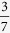 (154°), 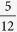 (150°), 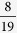 (152°)). This regular oscillatory behaviour is important because it is why the cost *u*(*α*_0_) has a local minimum at 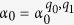 in Equation (3). In fact, the condition 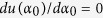 requires that *α*_0_ be equal to the numerical average of the resulting fractions 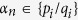, which is equivalent to saying that there should be no net angular shift between the two ends of the stem. The special angles 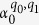 have this desirable property.

This model incorporates the plant’s specific features only through *l*_*n*_ in Equation (4). The relative depths of the local minima of the cost *U* depend on the lower limit of *l*_*n*_, whereas its fine structure depends on the upper limit of *l*_*n*_. For example, the result excluding the contributions from the first two leaves *n* = 1 and 2 is shown as the bottom thin purple line in Fig. 2. In special cases, other minima are as low as the golden angle (absolute minimum). In any case, however, the cost is globally minimized at the golden angle.

## Additional Information

**How to cite this article**: Okabe, T. Biophysical optimality of the golden angle in phyllotaxis. *Sci. Rep.* **5**, 15358; doi: 10.1038/srep15358 (2015).

## Supplementary Material

Supplementary Information

## Figures and Tables

**Table 1:** Divergence angles and corresponding sequences of phyllotaxis fractions.

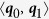	Divergence angle		Sequence		Species*
	*α*_0_	(°)	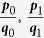	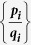	
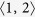	0.382	137.5	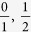	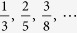	Predominant. Among others, 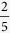 followed by 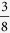 are the most common (pp.262–297 of ref. 2). Norway spruce *Picea abies* (cone)^2^ 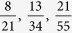 ; *Populus deltoides*^3^ 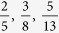 ; pineapple *Ananas comosus*^29^ 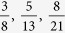 ; and *Helianthus annuus*^19^ 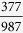 .
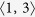	0.276	99.5	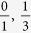	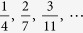	A typical anomalous sequence. *Cunninghamia lanceolata*^9^; *Sedum sexangulare*^2^ 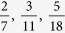 ; *Sedum reflexum* 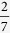 17, 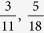 2; *Dipsacus sylvestris*^6^; and *Cedrus deodara*^22^.
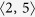	0.420	151.1	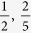	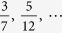	*Betula alba*; *Aloe spiralis* 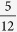 ; *Corylus tubulosa* 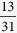 2; *D. sylvestris*^6^; and *Cephalotaxus drupacea*^21^.
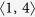	0.217	78.0	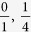	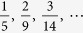	*S. reflexum* 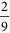 ; *Lycopodium reflexum*^2^ 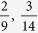 ; *D. sylvestris*^6^; *Cephalotaxus drupacea*; and *Cupressus macrocarpa*^25^.
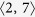	0.439	158.1	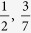	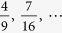	*Crinum americanum*^2^ 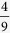 †; *Veronica longifolia*^2^ 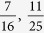 ; and *Musa sapientum*^30^ 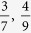 . ‡
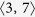	0.296	106.4	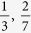	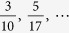	*Pothos* sp.^11^ and *Abies balsamea*^31^.
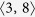	0.367	132.2	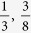	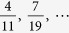	*Musa* bracts; *Agave americana* 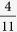 ; and *Grimmia leucophaea* 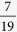 2.
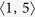	0.178	64.1	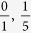	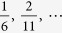	*Lycopodium rigidum*^2^ 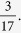
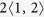		137.5/2		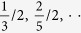	A typical anomalous sequence. *D. sylvestris*^2^,^6^; *Cephalotaxus fortunei*^22^; and *C. drupacea*^9^.
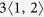		137.5/3		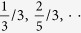	*Plantago media*(bracts)^2^ and *Araucaria excelsa*^22^.

^*^These are selected samples.

^†^Braun allotted this to an unusual sequence converging to 1/2 (see the short paragraph below Equation (3)).

^‡^Braun noted 3/7 for *Musa sapientum* and *rosacea*^2^. Knowing a fraction alone is not sufficient to infer the sequence to which it belongs, i.e., 〈2, 5〉 or 〈2, 7〉.

**Table 2:** Numbers of observed sequences.

Species	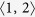	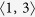	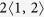	total
*P. Picea*^2^	1000	11	20	1000
*P. Abies*^2^	117	1	2	120
*P. abies*^32^	224	3	1	228
*P. sylvestris*^2^	>100	0	0	>100
*P. Strobus*^2^	54^*^	0	0	54
*P. Larix*^2^	41	0	0	41
*P. pendula*^2^	16	1	0	17
*P. alba*^2^	37	2	0	39
*Betula alba*^2^	46	0	6	54
*C. drupacea*(main)^21^	101	0	160	266^†^
(side shoot)	24	0	72	190^‡^
*Dipsacus sylvestris*^2^	0	0	50	50
*Dipsacus sylvestris*^6^	15	2	272	350^§^
*Plantago major*^2^	79	0	1	80
*Sequoia sempervirens*^33^	19	2	1	22
*Helianthus annuus*^23^	133	6	0	141
*Helianthus annuus*^24^	262	46	9	319
*Abies balsamea*^31^	3000	81	77	3200

Species names are presented as they appear in the cited references. ^*^Including 3 for 7/18, 1 for 11/29 and 1 for 12/31, which are not strictly 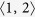. Similar notes shall apply to other cases. ^†^5 for 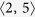. ^‡^94 for 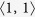. ^§^16 for 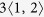.
