# Supplementary material for: Biophysical optimality of the golden angle in phyllotaxis
Source: Sci Rep. 2015 Oct 16;5:15358. doi: 10.1038/srep15358 (PMC4607949; doi:10.1038/srep15358)
Supplement: Supplementary Information [file srep15358-s1.pdf]

**Supplementary Information** for

# Biophysical optimality of the golden angle in phyllotaxis

Takuya Okabe (okabe.takuya@shizuoka.ac.jp)

Supplementary Note

Supplementary Figures S1-S4

**Supplementary Note**

A Fibonacci sequence derives from a pair of whole numbers $p_0$ and $p_1$ by the recursion formula

$$p_{i+2} = p_{i+1} + p_i. \qquad \text{(S1)}$$

An explicit form of the $i$-th term,

$$p_i = \frac{p_0\tau^{-1}+p_1}{\tau+\tau^{-1}}\tau^i + \frac{p_0\tau-p_1}{\tau+\tau^{-1}}(-\tau)^{-i}, \qquad \text{(S2)}$$

is established by noting that (i) it holds true when $i = 0$ and 1 and (ii) it obeys (S1) because the golden ratio $\tau$ defined by $1{:}\,\tau = \tau - 1{:}\,1$ satisfies

$$1 + \tau = \tau^2$$

and

$$1 + (-\tau)^{-1} = (-\tau)^{-2}.$$

Similarly,

$$q_i = \frac{q_0\tau^{-1}+q_1}{\tau+\tau^{-1}}\tau^i + \frac{q_0\tau-q_1}{\tau+\tau^{-1}}(-\tau)^{-i} \qquad \text{(S3)}$$

for

$$q_{i+2} = q_{i+1} + q_i. \qquad \text{(S4)}$$

Equations (S2) and (S3) give Equation (3) in the text, i.e.,

$$\alpha_0^{q_0,q_1} \equiv \lim_{i\to\infty}\frac{p_i}{q_i} = \frac{p_0+p_1\tau}{q_0+q_1\tau}. \qquad \text{(S5)}$$

Moreover, (S2), (S3) and (S5) give

$$q_i\alpha_0^{q_0,q_1} - p_i = \frac{p_0q_1-p_1q_0}{q_0+q_1\tau}(-\tau)^{-i+1}. \quad \text{(S6)}$$

For $i = 0$ and 1,

$$q_0\alpha_0^{q_0,q_1} - p_0 = -\frac{p_0q_1-p_1q_0}{q_0+q_1\tau}\tau, \qquad \text{(S7)}$$

and

$$q_1\alpha_0^{q_0,q_1} - p_1 = \frac{p_0q_1-p_1q_0}{q_0+q_1\tau}. \qquad \text{(S8)}$$

Subtracting (S7) from (S8),

$$(q_1 - q_0)\alpha_0^{q_0,q_1} - (p_1 - p_0) = \frac{p_0 q_1 - p_1 q_0}{q_0 + q_1 \tau} \tau^2. \qquad \text{(S9)}$$

Equations (S6) and (S9) give

$$q_i \alpha_0^{q_0,q_1} - p_i = \frac{(q_1 - q_0)\alpha_0^{q_0,q_1} - (p_1 - p_0)}{(-\tau)^{i+1}},$$

which is Equation (7) in the text.

Equations (S1) and (S4) give

$$q_{i+2} p_{i+1} - q_{i+1} p_{i+2} = -(q_{i+1} p_i - q_i p_{i+1}),$$

or

$$q_i p_{i-1} - q_{i-1} p_i = (-1)^{i-1}(q_1 p_0 - q_0 p_1).$$

For $p_i/q_i$ to be an irreducible fraction,

$$q_1 p_0 - q_0 p_1 = \pm 1.$$

Consequently, the limit divergence angle in Equation (3), or (S5), is specified by the initial denominator pair $\langle q_0, q_1 \rangle$ (the numerator pair of $p_0$ and $p_1$ is uniquely determined by $q_0$ and $q_1$).

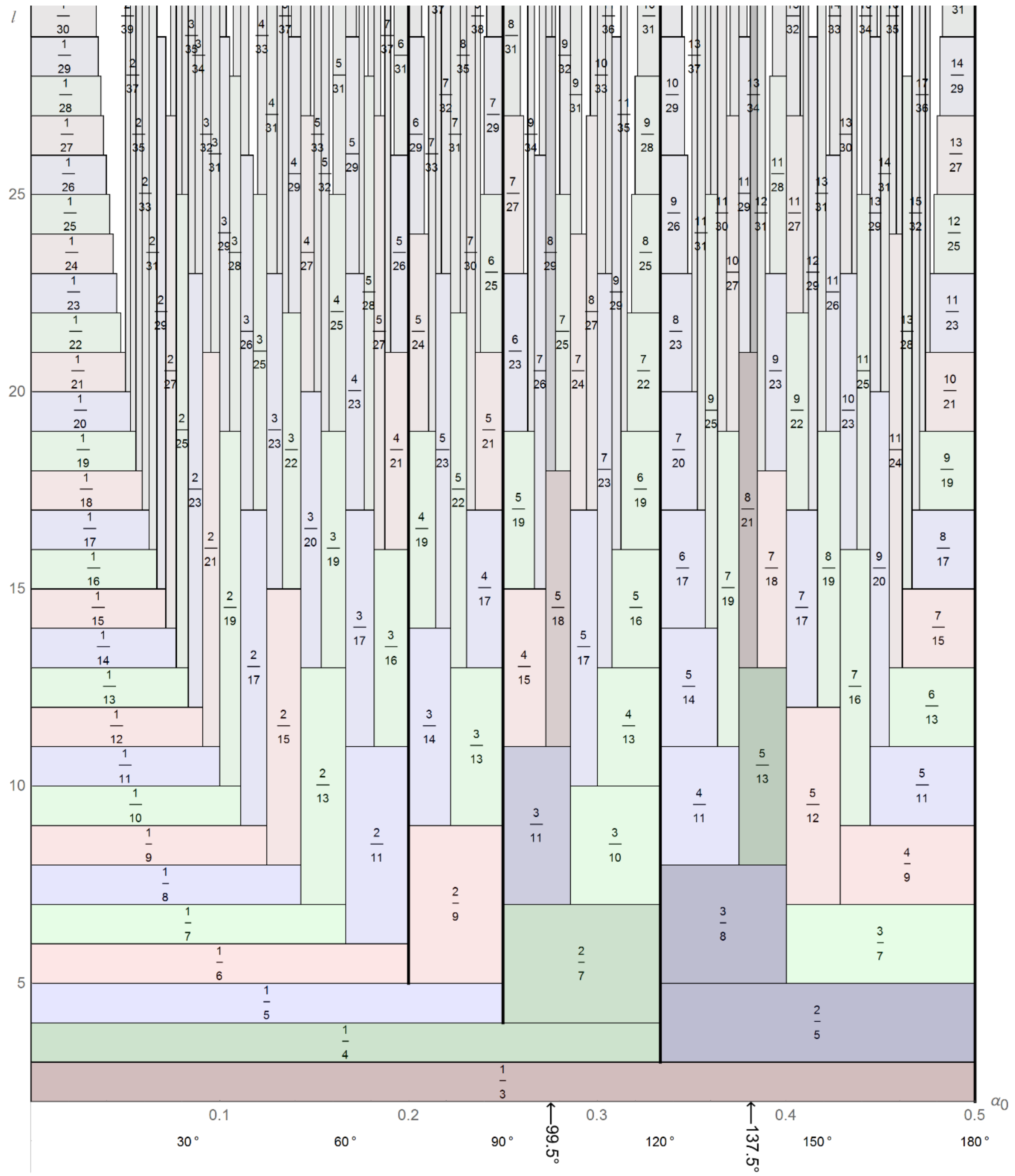


**Supplementary Figure S1 | The resulting phyllotaxis fraction depends on the initial arrangement.** This diagram shows the range of values of the initial divergence angle $\alpha_0$ and leaf-trace length $l$ for a given phyllotaxis fraction $\alpha$, i.e., this is a map of phyllotaxis fraction $\alpha(\alpha_0, l)$. The fraction $\alpha = 3/8$ is obtained for $0.33 < \alpha_0 < 0.40$ and $5 \leq l < 8$, as shown in Fig. S2b. The diagram contains all of the conceivable fractions between 0 and 1/2, which are broadly classified by thick vertical lines at $\alpha_0 = 1/n$ (shown for $n = 2,3,4,5$).

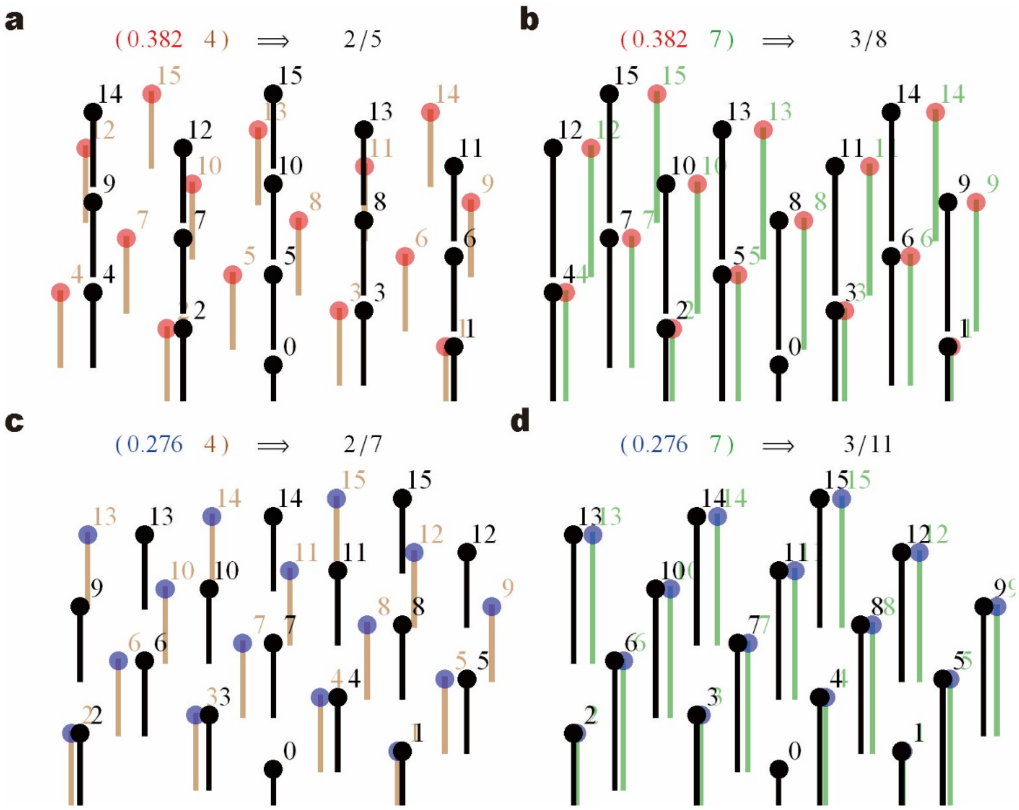


**Supplementary Figure S2 | The resulting phyllotaxis fraction depends on the initial arrangement.** (a,b) Initial patterns with the same divergence angle $\alpha_0 = 0.382$ (137.5°) and different lengths $l = 4$ (a) and 7 (b) result in different patterns of $\alpha = 2/5$ and $3/8$, respectively. (c,d) Similarly, $\alpha = 2/7$ (c) and $3/11$ (d) result from similar patterns with $\alpha_0 = 0.276$ $(99.5°)$. The resulting phyllotaxis fraction $\alpha(\alpha_0, l)$ is shown in Supplementary Fig. S1.

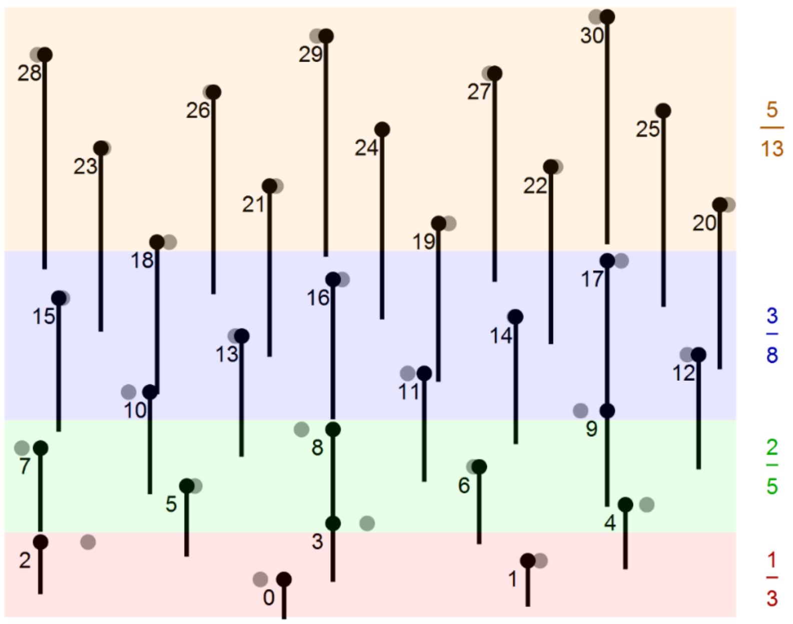


**Supplementary Figure S3 | The resulting phyllotaxis fraction depends on the initial arrangement.** A theoretical pattern modelled after Larson's diagram in Fig. 1d. Grey points represent an initial arrangement with $\alpha_0 = 0.382$, which are secondarily shifted to the positions of black points. The coordinates $(x_n, n)$ of a black point with a line of length $l_n$ are given by Equations (4), (5) and (6) for $\alpha_0 = 0.382$. From the bottom to the top, the phyllotaxis fraction $\alpha_n$ changes from 1/3 $(n = 1,2)$ through 2/5 $(3 - 8)$ and 3/8 $(9 - 17)$ to 5/13 $(18 - 30)$.

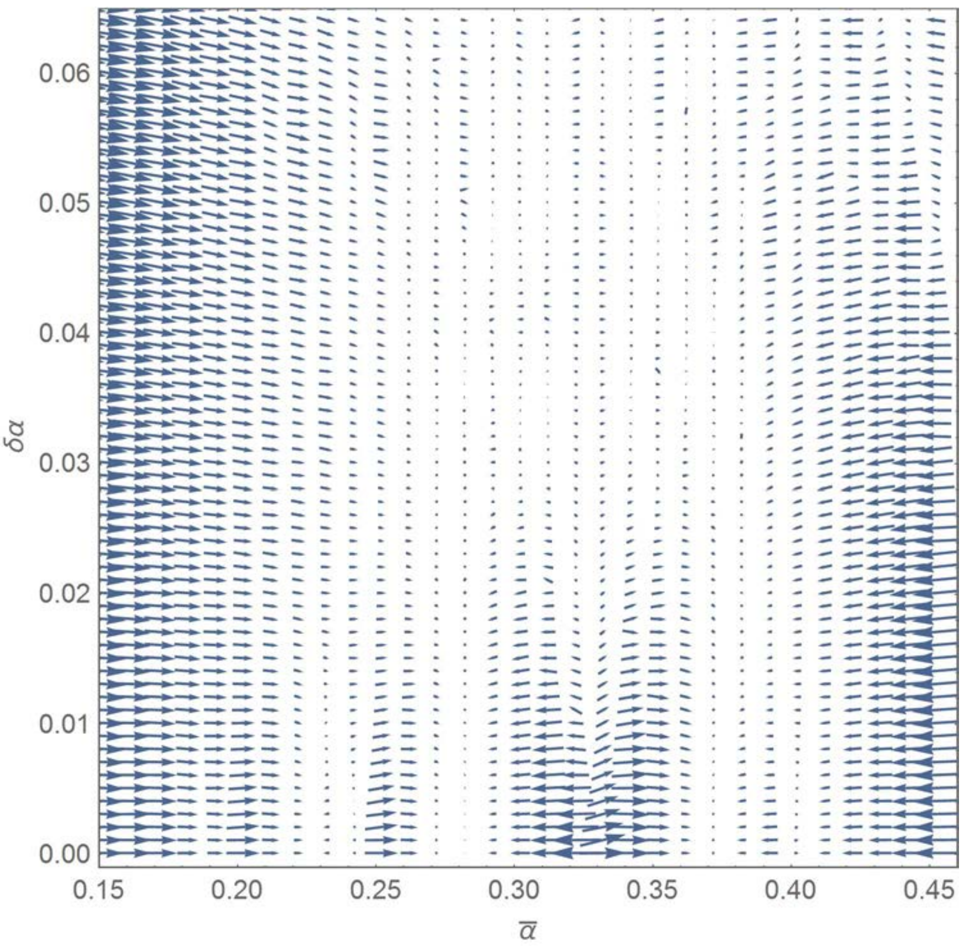


**Supplementary Figure S4 | Evolutionary trajectories of** $(\overline{\alpha},\ \delta\alpha)$**.** A vector field plot of $(\partial U/\partial\overline{\alpha}, 0.05\partial U/\partial(\delta\alpha))$.
